# B-value variations in the Central Chile seismic gap assessed by a Bayesian transdimensional approach

**DOI:** 10.1038/s41598-022-25338-4

**Published:** 2022-12-15

**Authors:** Catalina Morales-Yáñez, Luis Bustamante, Roberto Benavente, Christian Sippl, Marcos Moreno

**Affiliations:** 1 Department of Civil Engineering, Universidad Católica de la Santísima Concepción, Concepción, Chile; 2 Department of Geophysics, Universidad de Concepción, Concepción, Chile; 3 National Research Center for Integrated Natural Disaster Management (CIGIDEN), Santiago, Chile; 4 Institute of Geophysics of the Czech Academy of Sciences, Prague, Czech Republic

**Keywords:** Geophysics, Seismology

## Abstract

The b-value can be used to characterize the seismic activity for a given earthquake catalog and provide information on the stress level accumulated at active faults. Here we develop an algorithm to objectively estimate variations of b-value along one arbitrary dimension. To this end, we employ a Bayesian transdimensional approach where the seismic domains will be self-defined according to information in the seismic catalog. This makes it unnecessary to prescribe the location and extent of domains, as it is commonly done. We first show the algorithm’s robustness by performing regressions from synthetic catalogs, recovering the target models with great accuracy. We also apply the algorithm to a microseismicity catalog for the Central Chile region. This segment is considered a seismic gap where the last major earthquake with shallow slip was in 1730. Our results illuminate the downdip limit of the seismogenic zone and the transition to intraslab seismicity. In the along-strike direction, low b-value coincides with the extent of locked asperities, suggesting a high-stress loading at the Central Chile seismic gap. Our results indicate the reliability of the Bayesian transdimensional method for capturing robust b-value variations, allowing us to characterize the mechanical behavior on the plate interface of subduction zones.

## Introduction

The Gutenberg–Richter law linearly relates the logarithm of the number of earthquakes above a certain magnitude with the magnitude, and the slope of the resulting straight line is known as the b-value. Spatial variations of b-values in seismicity catalogs have been associated with different stress levels^1–3^, fluid processes^4,5^ as well as geological structures^6,7^. Moreover, it has been shown that b-values can discriminate between foreshock and aftershock activity^8–11^, and the temporal variation of b-values has been used for earthquake hazard estimation^12,13^. Given this breadth of potential applications, it is of high importance to robustly retrieve and characterize b-values as well as their changepoints (i.e. the places where the b-value varies). Most current methods map the b-value by fixing a spatio-temporal window of the seismic catalog (i.e. binning), and the b-value itself is calculated using optimization methods like maximum likelihood estimation^14,15^. While this is a standard procedure nowadays, choosing too small spatial and/or temporal bins can lead to insufficient sample sizes for b-value computation, which can lead to results that are difficult to interpret. Even when using large seismicity catalogs, attempts to achieve high spatiotemporal resolution often pay the price of decreased robustness of b-value estimates^4,16^. Moreover, expert choices such as the utilized mathematical formalism, sampling details or the choice of bin volumes^17–19^ as well as a number of biases frequently contained in earthquake catalogs^20^ can significantly affect obtained b-value distributions. Consequentially, it is hard to robustly determine when or where b-values change, and whether such an imaged change is only bias or artifact.

We here present a method to robustly determine one-dimensional b-value changepoints that does not rely on any spatiotemporal binning or smoothing. Instead, the algorithm solely relies on the seismicity catalog data, which is achieved with Bayesian transdimensional inference to simultaneously perform model selection and retrieve the b-value. Few authors have used Bayesian inference approaches to obtain the b-value and its changes. Kamer and Hiemer^21^ compute the changes using the Bayesian Information Criterion. They implement a data-driven tessellation based on Voronoi cells. Fiedler et al.^16^ employ a model selection methodology using the Bayes’ factors with an iterative algorithm to retrieve the b-value and its changes. In both model selection approaches, results from the fixed dimension problem are compared under a statistical criterion to assess consistency with the data. In contrast, in our transdimensional application, a posterior of variable dimension is directly sampled, performing the model selection naturally. To our knowledge, the present study is the first application of trans-dimensional inversion with the rjMCMC sampling methodology to estimate b-value variations. In the following sections, we first introduce our approach, then demonstrate its robustness by showing an application to two synthetic experiments with spatial changes. The first of these is simple, so that an analytical result for the model selection problem is available, the other is more realistic and hence more complex. Lastly, we apply our approach to a microseismicity catalog from Central Chile to characterize the seismic productivity of the seismic gap between the 2015 Illapel earthquake ($M_w$ = 8.3)^22^ and the 2010 Maule earthquake ($M_w$ = 8.8)^23^. We then compute the b-value variations with depth and latitude for the segment.

## Methods

The Gutenberg–Richter law^24^ can be expressed as:

1 $$\begin{aligned} \log N (m) = a - b (m - m_c), \end{aligned}$$

where *N* is the number of events in the catalog with a magnitude of at least *m*; $a=log(N(m_c))$, with $m_c$ being the detection threshold. *b* is the straight line slope that describes the proportion of large and small events, known as the b-value. The Gutenberg–Richter law is valid for catalogs that fulfill Eq. 1, which is generally the case for catalogs of independent events (i.e. background seismicity). Therefore, for a given b-value, the probability density function of occurrence of an earthquake with magnitude m is given by:

2 $$\begin{aligned} f(m) = \beta \ \exp (-\beta (m - m_c)) \end{aligned}$$

for all $m \ge m_c$, where $\beta = b \ln (10)$. Neglecting correlations among the event magnitudes, the joint probability density function *p* (PDF) of the magnitude $m_i$ for N events given $\beta$ corresponds to

3 $$\begin{aligned} p(\textbf{m}|\beta ) = \beta ^N \prod _{i=1}^N \exp (-\beta (m_i - m_c)). \end{aligned}$$

We are interested in assessing whether a given seismicity catalog can be described, with statistic significance, by one or more b-values along one arbitrary physical dimension (e.g., time, depth, latitude, longitude). Thus, if we call *k* the number of b-values required to explain the catalog, we are interested in estimating both $\beta _i$ and *k*, where $\beta _i$ corresponds to the i-th portion of the catalog.

We address this problem within a Bayesian inference framework. Bayesian inference is focused on characterizing the posterior probability density function (PDF) of specific model parameters given a set of measurements (the data). Since results are given in terms of the posterior PDF, this statistical methodology naturally allows to find the solution (e.g., a set of optimal parameters) and its uncertainty in inverse problems, intrinsically following the principle of parsimony. This principle states that where different models explain the observations similarly well, simple models will be preferred over complex ones.

Thus, we seek the posterior distribution (or values) of $\beta$ following a transdimensional Bayesian inference approach^25–27^,

4 $$\begin{aligned} p(\varvec{\beta },k|\textbf{m}) = \frac{p(\textbf{m}|\varvec{\beta },k)p(\varvec{\beta }|k)p(k)}{p(\textbf{m})}, \end{aligned}$$

where the symbol | (vertical line) represents a conditional probability. $p(\varvec{\beta },k|\textbf{x})$ describes the joint PDF for a set of $\varvec{\beta }$ and its dimension *k* given the data $\textbf{x}$ and is known as the *posterior*. $p(\textbf{x}|\varvec{\beta },k)$ is the *likelihood* of $\textbf{x}$ for a fixed dimension *k* and vector $\varvec{\beta }$. $p(\varvec{\beta }|k)$ represents the information of the parameters given the dimensions and *p*(*k*) the *prior* of the model parameters, which represents the knowledge that we have on some characteristic before measuring the data ${\textbf {x}}$. It is important to highlight that Bayes’ theorem allows an update of the prior considering the likelihood, which contains the data information^28^. If the data were poorly informative, this would be reflected in the posterior; the less informative the data, the more similar the posterior and prior PDF will be. $p(\textbf{x})$ is the marginal likelihood of the data known as the *evidence*. The evidence is not usually computed explicitly in inverse problems in geophysics, and it is treated as a normalization constant. However, the evidence has a key role in selecting the appropriate model between competing theories or parameterization choices in an inverse problem. In fact, Sambridge et al.^26^ show that the transdimensional posterior (Eq. 4), is related to the posterior for the fixed dimension problem and its respective evidence.

Frequently, the posterior PDF is not trivial, and its main statistics are not available analytically. In this case, the posterior PDF can be characterized via a sampling method. Such methods (as the name indicates) produce posterior PDF samples. There are several sampling methods, the most popular being Markov Chain Monte Carlo (MCMC). However, by incorporating the number of unknown model parameters as one of the unknowns of the inverse problem, the usual MCMC methods for sampling the posterior are inadequate, since they assume that all parameters in a model have exactly the same meaning in each sample; this is clearly not true for a trans-dimensional problem. To solve this issue, the transdimensional problem relies on the reversible jump Markov chain Monte Carlo (rjMCMC)^25^, which allows simultaneous sampling in different models and parameters. In the present work, we will use an implementation of the reversible jump Markov chain Monte Carlo sampling method (rjMCMC) as provided by the iEarth group (http://www.iearth.edu.au). This methodology corresponds to a robust Bayesian model selection framework, which has the advantage that it naturally follows the parsimony principle^26^. This means that unnecessarily complex solutions will be discarded, favoring simple solutions which still fit the data, with statistical significance. The rjMCMC algorithm will intrinsically draw more samples from models with high statistical significance. In particular, the algorithm would try a range of different models, and the models with more samples correspond to the ones preferred by the algorithm.

The final solution will be expressed in terms of the posterior. In order to process the solution, we need to remove its initial samples (the “burn-in stage”) to avoid bias caused by the initial models in the random walk^26^. To check whether the solution has converged or needs more samples, we compute the acceptance rate as the ratio between the accepted and the proposed models. In general, an acceptance rate close to or larger than 23.4% shows that the Metropolis algorithm worked correctly. This value may, however, change depending on the application^29^. The post-burn-in samples of the solution carry all the necessary information. From those samples, we select the ones with the most likely *k*, that is, the *k* value which is most frequently sampled. In turn, among those samples, we choose the best solution as the sample with the lowest misfit.

## Synthetic experiments

To evaluate the performance of our algorithm, we run a number of simulations employing synthetic seismicity catalogs with prescribed discontinuities in the b-value. We first present a simple case containing only one b-value change, which allows us to compare the results with an analytical result based on the conjugated prior technique. We also present a second case with a larger number of variations, for which analytical solutions become computationally too demanding. Thus, in this case, only comparison with the target model is performed. The synthetic catalogs were computed based on Eq. 2. We then use the analytic solution and rjMCMC to invert the synthetic catalog and retrieve the positions of the different segments.

**Figure 1:**
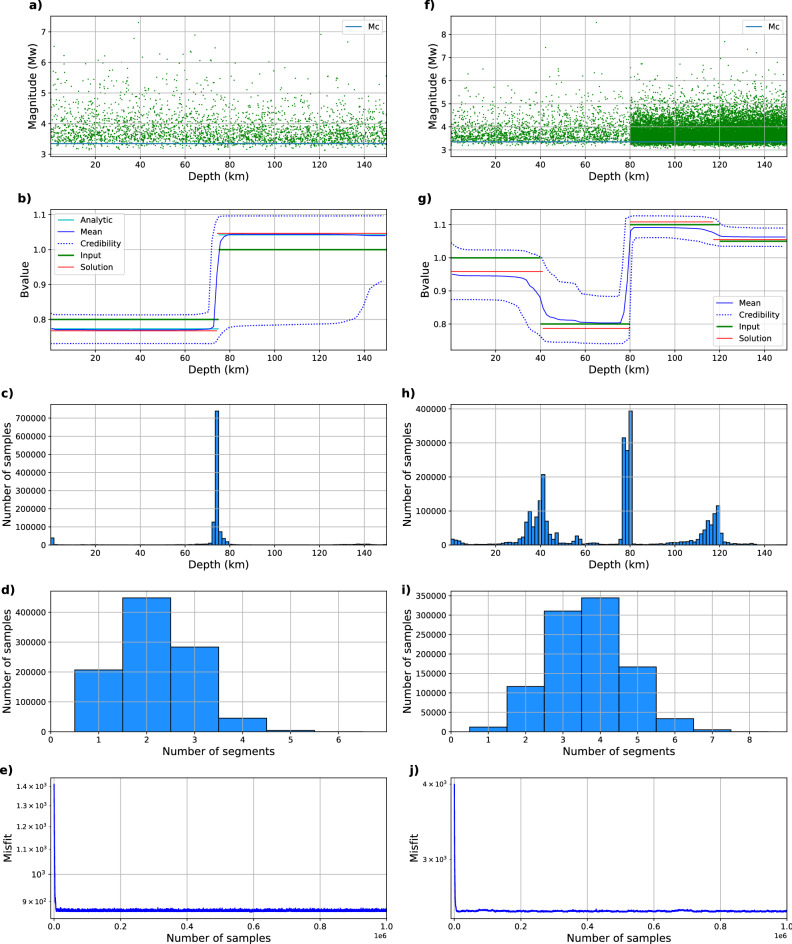
Summary of two synthetic tests. The left column shows results from a simple case featuring two segments of b = 0.8 and b = 1.0, with a homogeneous a-value (3.2, i.e. $\sim$ 1500 events per segment). The right column shows results from a more complex scenario involving four segments with different b (1, 0.8, 1.1, 1.05) and a-values (3, 3, 4, 4; i.e. 1000 or 10,000 events per segment). In both cases, random noise with a standard deviation of 0.1 was added to the magnitudes. (**a**) and (**f**) synthetic earthquake catalogs generated using the two and four sets of earthquakes with different b-values. (**b**) and (**g**) b-value input (green), analytic solution (cyan), solutions obtained using rjMCMC: mean (blue), the best fit (red), and credibility interval (dotted blue). (**c**) and (**h**) Histograms that represent the amount of samples that present a change in b-value at a certain depth. (**d**) and (**i**) Histograms that represent the amount of samples of models with a specific number of segments. (**e**) and (**j**) Evolution of solution misfit.

For each example, we run 1,000,000 samples and remove the first 10,000 to avoid bias from the initial model (burn-in stage). The solutions (presented in Fig. 1b) show the mean b-value of the samples with a credibility interval of 95%. The preferred model is the model with the number of segments that has the most samples (that is, ${\hat{k}}$). We also show the best solution obtained by selecting the sample with the lowest misfit among the models with the most probable number of parameters ${\hat{k}}$.

### Analytical method

Sambridge et al.^26^ show that the trans-dimensional posterior sampling using rjMCMC can be replicated from fixed dimension sampling methods, although this can be inefficient for some applications. Such a strategy provides an independent method that is useful for testing purposes. We thus compare the solutions retrieved with the rjMCMC and the evidence from the Bayes theorem as suggested by Sambridge et al.^26^.

To estimate the analytic solution, we use a strategy of Bayesian inference called conjugate prior. The conjugate prior is a PDF that together with the likelihood results in a posterior of the same family of distributions as the prior. This approach is advantageous only when the likelihood has an analytic solution, which is the case in the present study. To obtain the b-value, the exponential distribution (Eq. 3) is used as a likelihood function. The conjugate prior of the exponential distribution corresponds to a Gamma distribution. Thus, by choosing:

5 $$\begin{aligned} P(\beta ) = \frac{1}{\Gamma (\alpha _0)\theta _0^{\alpha _0}}\beta ^{\alpha _0-1} e^{-\beta /\theta _0}, \end{aligned}$$

and employing the likelihood in Eq. 3, the posterior is given by:

6 $$\begin{aligned} P(\beta |\textbf{m}) = \frac{P(\textbf{m}|\beta ) P(\beta )}{P(\textbf{m})} = \frac{1}{P(\textbf{m})} \left( \beta ^N \prod _{i=1}^N \exp (-\beta (m_i - m_c))\right) \ \left( \frac{1}{\Gamma (\alpha _0)\theta _0^{\alpha _0}}\beta ^{\alpha _0-1} e^{-\beta /\theta _0}\right) \propto \beta ^{\alpha -1} e^{-\beta /\theta } \end{aligned}$$

where $\alpha = \alpha _0 +n$ and $\theta ^{-1}= 1/\theta _0 +n \overline{m}$, with *n* the number of samples of the segments, $\overline{m}$ the mean of the magnitudes (with $m_c$ subtracted) of the corresponding catalog, and $\alpha _0$ and $\theta _0$ parameters of the gamma function which we choose to be 1.5 and 5.0, respectively, resulting in the fairly uninformative prior depicted in Figure S1. The proportionality symbol indicates that the normalization constant has been omitted.

Since the resulting posterior is a Gamma distribution, whose normalizing constant is $1/[\Gamma (\alpha )\theta ^{\alpha }]$, we can set the evidence $P(\textbf{m})$ to:

7 $$\begin{aligned} P(\textbf{m}) = \frac{\Gamma (\alpha )\theta ^{\alpha }}{\Gamma (\alpha _0)\theta _0^{\alpha _0}}. \end{aligned}$$

To find the position where the b-value changes, we use the maximum evidence principle^30^ as our model selection tool. This means that the evidence will be evaluated for each model, and the one maximizing the evidence will be selected. In our problem, models can have different amount and extent of segments with a characteristic b-value. By this method, we can select the simplest model which is consistent with the data.

### Simple case: one discontinuity

For a comparison with the analytical solution, we generate a synthetic seismicity catalog with one discontinuity in the b-value, i.e. two segments. The segments have b-values of 0.8 and 1.0 and a-values of 3.2 (equivalent to $\sim$ 1500 events per segment). We use these values to evaluate the algorithm’s capacity to retrieve a b-value change of 0.2. The minimum magnitude of the earthquakes is $M_w = 3.35$. To create the synthetic catalogs, we used Eq. 2, and we add noise to the magnitude data from a random normal distribution with a standard deviation of 0.1. The depth value for each event is generated using a random uniform distribution between 0 and 150 km.

Figure 1a visualizes magnitudes and depths of events in the synthetic catalog. Figure 1b shows the solutions obtained using the analytical method and the rjMCMC approach. Both solutions retrieve b-values close to what was used to create the synthetic data, with differences in the range of 0.04, for each segment. These differences result from a combination of the added noise, uncertainties due to the relatively low amount of utilized data, as well as from the method itself. The rjMCMC algorithm shows an acceptance rate of 40%, which demonstrates the convergence of the solution. Figure 1c shows the number of times that rjMCMC selects a model with segment limits at each depth for the selected model. We see that the vast majority of models feature a change in the b-value at a depth of 75 km, which is the value used to create the synthetics. Figure 1d shows the distribution of segment numbers (models) that make up the different samples. It can be observed that the preferred model corresponds to samples with two segments. The low retrieved misfit shown in Fig. 1e and the similarity with the analytical solution demonstrates the reliability of the rjMCMC methodology.

### Complex case: several discontinuities

Solving the problem analytically seems to be a simple path to resolve the b-value inversion. However, when more than two or even an indeterminate number of segments is present, the problem becomes more complex and computationally expensive compared with the rjMCMC approach, since it involves a grid search over a (potentially large) multidimensional space. Also, the conjugated prior approach is valid only for the exponential likelihood in Eq. 2, which can be an unwanted restriction in some applications.

To test if the algorithm can retrieve more complex scenarios, we created a synthetic dataset with multiple discontinuities in depth. We use four different segments with b-values of 1, 0.8, 1.1 and 1.05, and a-values of 3, 3, 4 and 4, which corresponds to 1000, 1000, 10,000 and 10,000 events, respectively. The minimum variation of the b-value is of the order of 0.05. As before, the minimum magnitude of the earthquakes is $M_w = 3.35$. The magnitude is computed using Eq. 2, adding noise from a random normal distribution with a standard deviation of 0.1. The depth value for each event is assigned using a random uniform distribution. We run 1,000,000 samples and remove the first 10,000 (burn-in stage). For the best sample, mean value and credibility interval, we use the same definitions as in the previous section.

Figure 1f–j summarizes our results. The acceptance rate corresponds to $\sim$ 40% of the samples. We found that the a-value has an influence on the retrievable b-value variations. As the a-value is linked to the number of earthquakes in a segment, we found that if we have a large number of earthquakes (10,000), even small variations of the b-value ($\sim$ 0.05) can be reliably retrieved; however, for small numbers of earthquakes (on the order of 1000), the algorithm only retrieves larger variations in the b-value on the order of 0.2. For additional synthetic tests that further explore the influence of the number of earthquakes per segment, the reader is referred to the Supplementary Material (Figures S2–S4). We also conducted a test with homogeneous b-value and different a-values, which shows that a-value changes should not affect the retrieved b-value changepoints when reasonable event numbers are used (Figure S4). Figure 1g shows the solutions obtained using rjMCMC, where it can be seen that target b-value and the solutions are similar. The largest difference between input model and retrieved solution is of the order of 0.05, and occurs where fewer events are present. It can be observed that when having fewer earthquakes, the credibility interval is wider than when more earthquakes are used. Figure 1h shows the variations of the b-value as a function of depth, where for most cases, the changepoint is clearly resolved, but where the algorithm explores several different solutions for its exact position. The changepoints used to create the catalog were located at depths of 40, 80 and 120 km; the retrieved values are 41, 80 and 117 km, showing the capacity of the code to find these changepoints. Figure 1i shows that the algorithm explores different models, where the preferred one is the model with four segments.

We find that the rjMCMC approach performs reliably for simple and complex cases, provided that the utilized catalog contains a sufficient number of earthquakes. In addition, it needs less a priori information than the analytical solution, which relies on the given Gamma distribution parameters. Furthermore, the information that can be obtained from the analytic solution is more limited than what the rjMCMC approach yields.

## Application to central Chile

The subduction of the Nazca beneath the South American tectonic plate produces large-scale earthquakes along the coast of Chile. In particular, the Central Chile margin (here defined between 29$^\circ$ S and 35$^\circ$ S) is bounded by the rupture zones of the 2015 Illapel earthquake ($M_w$ = 8.3)^22^ in the north and 2010 Maule earthquake ($M_w$ = 8.8) rupture^23^ in the south. This area was ruptured by the $M_w$ = 8.4 Valparaiso earthquake in 1906 and then partially broken by M 8 events in 1971 and 1985. The surface effects produced by the 1906 event suggest that its rupture zone was deeper (within zones B and C of Lay et al.^31^), and probably the shallow part of the seismogenic zone was not involved. Surface effects of the 1985 earthquake indicate only a partial release of the accumulated slip at shallow depths since 1730, when a giant tsunamigenic earthquake with a magnitude in the range of $M_w$ 9.1–9.3 occurred^32^, which is the most significant historical event in this area. The large seismic gap in the region calls the interest of scientists, and several configurations of seismic asperities that may define the extent of future earthquakes have been proposed^33,34^. It is for this that we apply the proposed methodology to central Chile. The seismic data used in this study correspond to a microseismicity catalog composed of close to 12,000 events between 2014 and 2018. Magnitudes in the catalog are reported as local magnitudes $M_l$^34^.

### Catalog processing

To make use of the catalog, we first homogenize the magnitudes to $M_w$. To convert the magnitudes from $M_l$ to $M_w$, typically regional analysis is needed^35^. We perform a regional transformation of the magnitudes by linear regression (Figure S5) between common events in the original microseismicity catalog and the GCMT database^36,37^. We convert events with $M_l < 6.5$ to $M_w$, while for $M_l > 6.5$ we use the $M_w$ values computed by the GCMT database. We then estimate the magnitude of completeness $m_c$, which is the magnitude from where the detection is reliable. Although $m_c$ may vary in time and space, we here compute a conservative estimate for $m_c$ for the whole catalog. For this, we use the algorithm of Mizrahi et al.^38^, which is based on calculating b-values for different $m_c$ values and the comparison between observed and theoretical cumulative magnitude distribution functions. We obtain that $m_c = 3.35$ for the complete catalog (Figure S6).

The methodology presented in this study is based on the joint probability shown in Eq. 3. Both this equation and the Gutenberg–Richter law require independence between events. The set of independent events corresponds to the background seismicity, which is composed of events that are neither precursors nor aftershocks of one another. To isolate the background seismicity, we declusterize the seismic catalog, relying on the implementation of ETAS (Epidemic-Type Aftershock Sequence)^38^ (further information in Text S1) to perform the declusterization.

**Figure 2:**
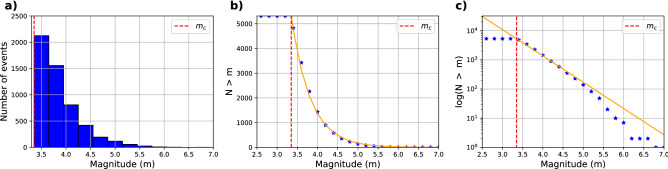
Solutions of completeness magnitude for the declusterized and filtered catalog. Figure (**a**) shows the frequency of the earthquakes as a function of magnitude. (**b**) The cumulative number of earthquakes (blue stars) and the Gutenberg–Richter equation that best fits the solution (orange); (**c**) shows the logarithmic version of (**b**).

**Figure 3:**
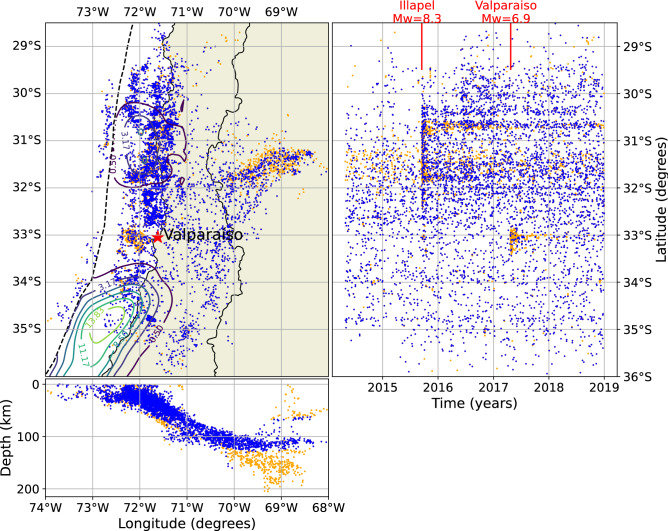
Spatial and temporal analysis of the seismicity catalog used in the present study. The colors indicate background seismicity (blue) and dependent events (orange). Dashed lines indicate the position of the trench, and solid lines trace slip contours of the 2015 Illapel earthquake ($M_w=8.3$)^22^ and the 2010 Maule earthquake ($M_w=8.8$)^23^. The red star indicates the position of Valparaíso. Red lines shows the position in time of the 2015 Illapel earthquake and the 2017 Valparaiso earthquake ($M_w=6.9$)^49^. This figure was created using Python 3.8.13^50^, Matplotlib 3.5.2^51^ and Cartopy 0.21.0^52^.

Finally, we select plate interface and intraslab earthquakes with a distance of < 30 km from the slab2.0 surface^39^, from the declusterized and homogenized catalog. From originally 12,000 events, 5272 remain after these selections. We recalculate $m_c$ for the declustered and filtered catalog, obtaining $m_c=3.35$ (the same as the initial catalog) and an overall b-value of 0.9 (Fig. 2). Figure 3 shows the original catalog, with colors indicating the background seismicity (or independent events) in blue and the dependent events in orange. The dependent events are predominantly aftershocks of the 2015 Illapel earthquake ($M_w=$ 8.3) and the 2017 Valparaiso earthquake ($M_w=$ 6.9).

### B-value in Central Chile

**Figure 4:**
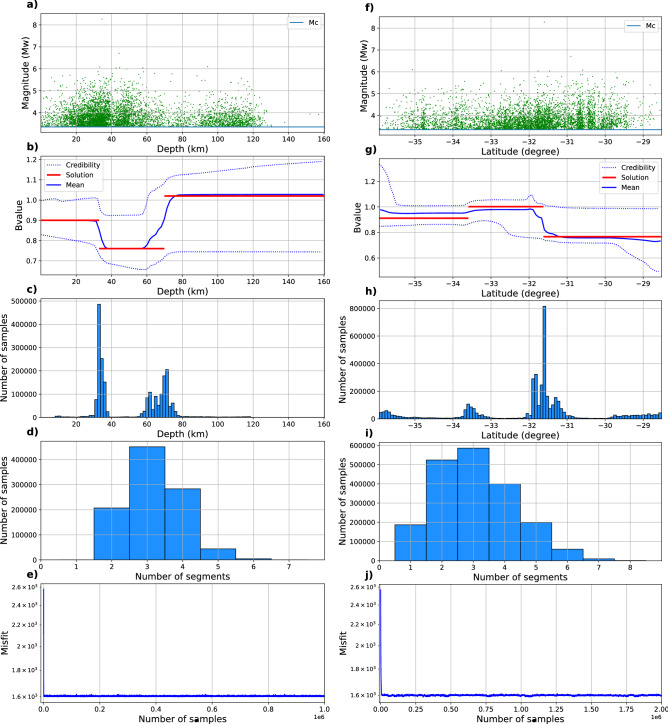
Applications of the rjMCMC to determine b-value variation as a function of depth (**a**–**e**) and latitude (**f**–**j**) for real data from Central Chile (5272 events in total). (**a**) and (**f**) Earthquake catalog as a function of the dependent variable. (**b**) and (**g**) Solutions obtained using rjMCMC: mean (blue), the best fit (red), and credibility interval (dotted blue). (**c**) and (**h**) Histograms representing the amount of samples that show b-value changepoints at a specific depth or latitude. (**d**) and (**i**) Histograms representing how many samples showed each number of segments. (**e**) and (**j**) Evolution of solution misfit.

**Figure 5:**
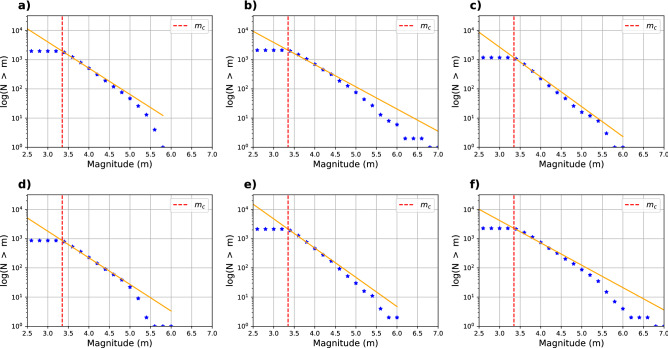
The figures show the Gutenberg–Richter law representation of the obtained b-value for each segment. Upper figures represent the solutions along strike, and lower figures correspond to the results along dip. The blue star represents the number of events larger than a magnitude *m*, the orange line corresponds to the Gutenberg Richter law computed with the obtained b-value, and the red dotted line represents the chosen $M_c$. (**a**), (**b**) and (**c**) correspond to the events from 0 to $\sim$ 33 km, from $\sim$ 33 to $\sim$ 70 km, and from $\sim$ 70 to $\sim$ 160 km depth, respectively. Subfigures (**d**), (**e**), and (**f**) correspond to the events between 35.94$^\circ$ to 33.59$^\circ$ S, 33.59$^\circ$ to 31.62$^\circ$ S and 31.62$^\circ$ to 28.5$^\circ$ S, respectively.

We compute the b-value variation as a function of depth and latitude independently. Figure 4 shows the results for both of the variables and Fig. 5 shows the fitting of the obtained b-values with the data in each segment. For the implementation as a function of depth, we draw 1,000,000 samples with a burn-in period of 10,000. The acceptance rate of the samples is 24%. The results are shown in Fig. 4a–e. We retrieve two changepoints of the b-value at approximately 30 and 70 km depth. The shallower segment has a b-value of 0.9, the middle segment has a b-value of 0.76, whereas a significantly higher b-value of 1.02 is obtained for the deeper segment. Figure 4c shows that the method explores various b-value changepoints located between 60 and 80 km depth; however, models with the change located at 70 km dominate in the solution, as shown in Fig. 4c. The large credibility interval is likely due to the comparatively low amount of data, as observed in the synthetic case, where regions with fewer earthquakes exhibited larger credibility intervals.

The observed changepoints of the b-value at $\sim$ 30 and $\sim$ 70 km depth could be linked to downdip segmentation of the plate interface ($\sim$ 30 km) as well as the onset of decoupling at the termination of the plate interface (typically located between 20 and 50 km depth^40^). Furthermore, the seismicity above and below about 60–70 km depth usually has different locations with respect to the subducted plate. At depths shallower than $\sim$ 60–70 km, the majority of earthquakes occur on or close to the plate interface, while the seismicity below consists of intra-slab earthquakes. As shown in Sippl et al.^41^ for Northern Chile, these different earthquake populations also show distinct b-values, with the plate interface exhibiting lower b-values (0.58 in Northern Chile) than intraslab populations (between 0.63 and 0.89 in Northern Chile). Likewise, Poulos et al.^13^ classify events shallower than 60 km as interplate and obtain systematic differences with intraslab events (> 60 km) in the North of Chile. They obtain b-values of 0.86 and 1.02 for the interplate and intraslab segments. The b-value estimates obtained in the present study follow the general trend in previous works, finding lower b-values (0.89 and 0.76) for the shallow segments (0–30 km and 30–70 km) and a higher b-value (1.02) for the deeper one (70–150 km). We acknowledge that our estimates of absolute b-values depend on the method for choosing $m_c$: when using the approach advocated in Herrmann and Marzocchi (2021)^42^, we retrieve a higher value for $m_c$, which would lead to somewhat higher absolute b-values.

For retrieving b-value changepoints as a function of latitude, we likewise draw 1,000,000 samples with a burn-in period of 10,000 samples. The acceptance rate is 27%. Figure 4f–j shows the results of the approach, which identifies the presence of three segments. The changepoints of the best solution are located at 33.6$^\circ$ S and 31.6$^\circ$ S, and the b-values of the three segments are 0.91, 1.00 and 0.76. The credibility interval shows large variability at the edges of the domain. This is likely due to the paucity of events, resulting in poorly constrained b-values there. Such artifacts are typical for transdimensional Bayesian inversion methods^43^, and arise due to the difficulty to constrain the solution in the vicinity of the sudden discontinuity of the domain border.

**Figure 6:**
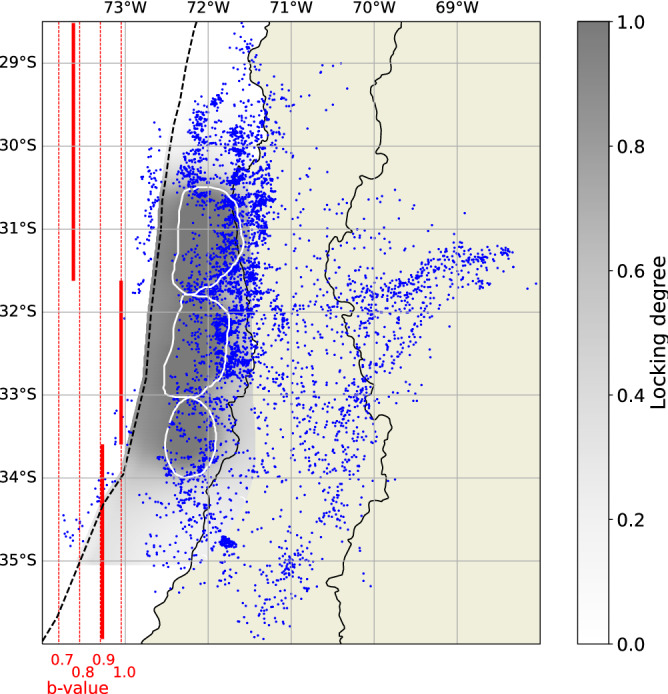
The map shows the b-values obtained in the present study in red, with the locking degree (greyscale) and the asperities (white line) proposed by Sippl et al.^34^. It also shows the seismicity used in the study (blue dots) and the trench (dashed line). This figure was created using Python 3.8.13^50^, Matplotlib 3.5.2^51^ and Cartopy 0.21.0^52^.

The segments we retrieve correlate well with the configuration of asperities proposed by Sippl et al.^34^ based on seismicity outlines. Figure 6 shows the asperities obtained by Sippl et al.^34^, with the declustered catalog and our b-value results. Our changepoints in b-value correlate well with two of the reported asperity boundaries; however, we do not identify the boundary around 32$^\circ$S. Previous studies along strike-slip faults^44,45^ as well as subduction megathrusts^46^ have shown that seismic asperities can be identified as regions of anomalously low b-value, which has been linked to the presence of elevated stress levels. However, it is controversial whether such signals are stable over long time periods. Here, we observe that adjacent potential asperities yield distinct b-values and thus create a signature in our changepoints that could be related to spatial variations in the stress level and rheology. However, we also acknowledge that including intraslab events as well as plate interface events into this search for changepoints in latitude may lead to results (b-values themselves as well as changepoint locations) that are not easy to interpret. The missing changepoint at 32$^\circ$S can be attributed to several causes, such as a similarity of the stress levels between adjacent asperities, an only very slight variation between their b-values, a too low number of used events, or the depths of the events, which as we show can produce changes in the b-value. Our results generally agree well with the global study of subduction megathrust earthquakes by Bilek et al.^47^, which indicates that almost all subduction zones show b-values that are lower than one, with megathrust areas usually featuring b-values between 0.7 and 0.8. Legrand et al.^4^ studied the b-value variation in time and space in Chile, and related b-values between 0.6 and 0.8 to seismic asperities and high shear stresses. The positive and negative variations in the b-value can be associated with different sources^48^. To determine which source (e.g., rheology, stress) is the one that dominates the solutions of the variation in the b-value obtained in this work, more studies and data are needed. However, it appears that the changepoints can to some degree illuminate the locked asperities reported in previous studies.

To ascertain that our results do not critically depend on the performed catalog declusterization, we ran additional inversions for a filtered but non-declusterized catalog as well as an “over-declusterized” catalog (see Supplementary Material, Figs. S8 and S9). We note that only the most significant b-value changes appear in all inversions, while the change-points slightly move. In contrast, the smaller variations are not consistently retrieved and may vanish or merge with others (further discussion in the Supplementary Material).

These comparisons provide a hint that our methodology may be capable of identifying the b-value signatures of different asperities in a real-world scenario, given that the input data feature sufficient quality and event numbers.

## Conclusion

In this work, we apply a Bayesian transdimensional methodology to a seismicity catalog of central Chile, showing the possibility of recovering b-value variations along-dip and along-strike. Additionally, the solutions along-strike show similarities with the extent of asperities proposed in previous studies, illustrating the possible utility of the algorithm in detecting strongly coupled areas of the megathrust. The results along-dip correlate with the transition from interplate to intraslab earthquakes.

To retrieve these solutions, we developed a Bayesian transdimensional approach with an rjMCMC sampler to estimate b-value changepoints in one dimension for independent events. This methodology allows determining the b-value without any arbitrary segmentation, where the data itself yields the number of changepoints. The methodology successfully retrieves b-values utilized to create synthetics, with solutions comparable to an analytical method. The synthetic tests help us estimate the methodology’s resolution as well as its dependence on the utilized number of events. The solutions converge and offer clear selections of the preferred models.

We have demonstrated the Bayesian transdimensional approach’s reliability, capability, and potential to resolve the problem of b-value variation in one dimension. Its principal benefit comes from the rjMCMC sampler, which allows to include the number of variations as a parameter to determine, obtaining the number of segments and their b-values simultaneously. While this work only illustrates the algorithm retrieving b-value variation along a single spatial dimension, obtaining variations with time can be treated similarly. Extending the algorithm to two-dimensional problems could resolve reliable delimitation of seismic domains; however, it requires a more complex spatial partitioning scheme, such as Voronoi cells. We do not explore such applications here but instead focused on simple applications to illustrate the method’s capabilities.

## Supplementary Information

Supplementary Information.

## Data Availability

Microseismicity catalog available through^34^. The implementation of the reversible jump Markov chain Monte Carlo sampling method (rjMCMC) is provided by the iEarth group (http://www.iearth.edu.au). The changes applied to the algorithm were minimal, and are plausible following the manuscript. In case guidelines are needed, please contact the authors (catalina.morales@ucsc.cl).
