# Supplementary material for: B-value variations in the Central Chile seismic gap assessed by a Bayesian transdimensional approach
Source: Sci Rep. 2022 Dec 15;12:21710. doi: 10.1038/s41598-022-25338-4 (PMC9755127; doi:10.1038/s41598-022-25338-4)
Supplement: Supplementary file 1 — Supplementary Information. [file 41598_2022_25338_MOESM1_ESM.pdf]

Supplementary information: B-value variations in the Central Chile seismic gap assessed by a Bayesian transdimensional approach

Catalina Morales-Yáñez[1,*], Luis Bustamante[2], Roberto Benavente[1,3], Christian Sippl[4], and Marcos Moreno[2]

[1]Department of Civil Engineering, Universidad Católica de la Santísima Concepción, Concepción, Chile.
[2]Departament of Geophysics, Universidad de Concepción, Concepción, Chile
[3]National Research Center for Integrated Natural Disaster Management (CIGIDEN), Santiago, Chile
[4]Institute of Geophysics of the Czech Academy of Sciences, Prague, Czech Republic
[*]catalina.morales@ucsc.cl

Analytical method

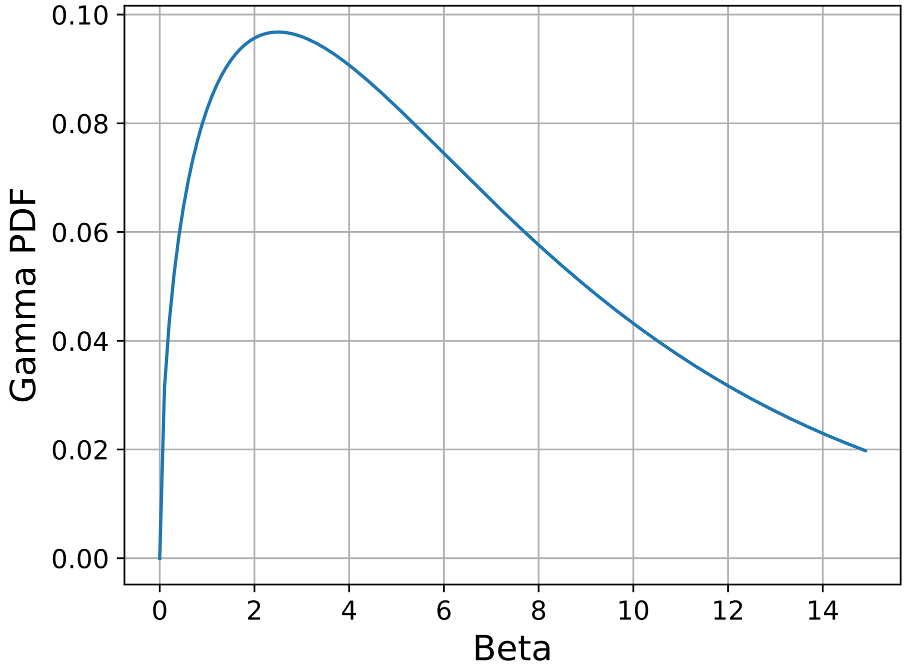

Figure S1. The Gamma PDF used to compute the analytical solution. α_0 and θ_0 constants from equation 6, of the gamma function, are 1.5 and 5.0, respectively, resulting in a fairly uninformative prior.

Synthetic tests

To explore the effect of the number of earthquakes on the solutions, we create synthetic catalogs with three segments and a constant a-value. For all the catalogs, the b-value remains the same, being 1.0, 0.8, and 1.1 for the shallow, middle, and deepest segments, respectively. Figure S2 shows the obtained solution for the catalog with an a-value corresponding to 500 events per segment, without (a-e) and with added noise (f-j). The retrieved b-values are 1.08, 0.75, and 1.04 for the example without noise, and 1.05, 0.75, and 1.14 for the synthetics with added noise. Figure S3 shows the obtained solution for a catalog with an a-value corresponding to 1000 events per segment, without (a-e) and with noise (f-j). The retrieved b-value here are 0.97, 0.79, and 1.06 for the example without noise, and 0.95, 0.78, and 1.2 for the case with added noise. We observe that the differences between the examples with and without noise are mostly minor and largely show larger uncertainty in the solutions for the noisy cases. Increasing the number of earthquakes narrows the credibility intervals and generally leads to retrieved values closer to the input values.

We also explore the effect of the a-value and b-value changes. We created a synthetic catalog with four segments to represent the solutions just with a-value changes and just with b-value changes. The changepoints are at 50, 100, and 150 km. For the first, second, third, and fourth segments, we have an a-value of 10000, 1000, 1000, and 3000 events and a b-value of 1.0, 1.0, 0.8, and 0.8, respectively. Figure S4 shows the solution for synthetics without (a-e) and with noise (f-j). Solutions show to well retrieve the b-values with a maximum difference of 0.02. The results also show that there is no effect in the a-value change point, showing that the algorithm is not sensitive to a-value changes while it is to b-value ones.

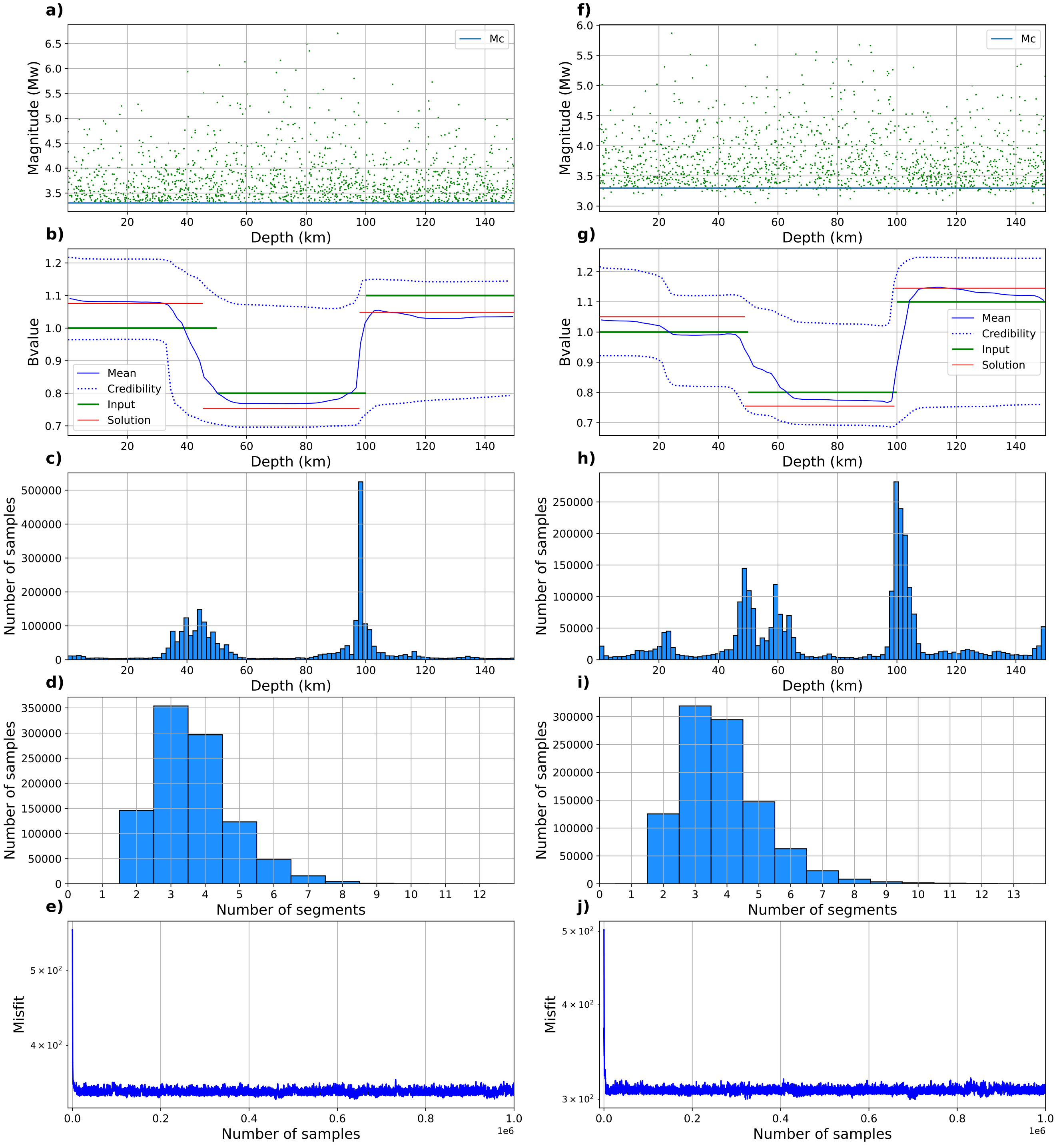

Figure S2. Summary of two synthetic tests using 500 events per segment and a scenario of three segments with different b-values. The left column shows results for a noise-free catalog, in the right column noise (standard deviation 0.1 as in the main article) was added to the magnitudes. a) and f) synthetic earthquake catalogs generated using two and four sets of earthquakes with different b-values. b) and g) b-value input (green), analytic solution (cyan), solutions obtained using rjMCMC: mean (blue), the best fit (red), and credibility interval (dotted blue). c) and h) Histograms that represent the amount of samples that present a change in b-value at a certain depth. d) and i) Histograms that represent the amount of samples of models with a specific number of segments. e) and j) Evolution of solution misfit.

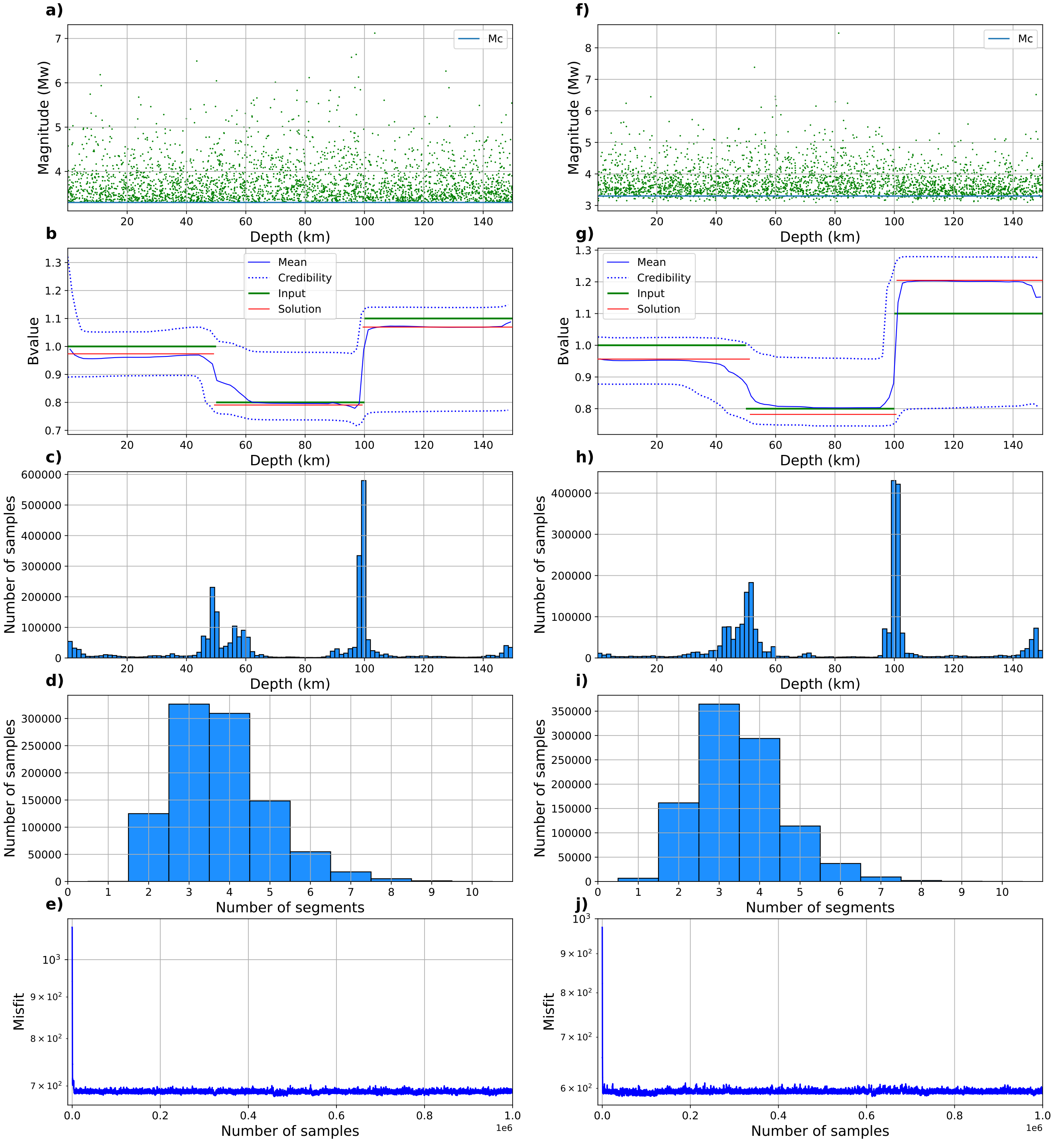

Figure S3. Summary of two synthetic tests that are identical to those in Figure S3, but with 1000 events per segment. Again, the left column shows results for a noise-free catalog, whereas in the right column noise was added to the magnitudes. a) and f) synthetic earthquake catalogs generated using two and four sets of earthquakes with different b-values. b) and g) b-value input (green), analytic solution (cyan), solutions obtained using rjMCMC: mean (blue), the best fit (red), and credibility interval (dotted blue). c) and h) Histograms that represent the amount of samples that present a change in b-value at a certain depth. d) and i) Histograms that represent the amount of samples of models with a specific number of segments. e) and j) Evolution of solution misfit.

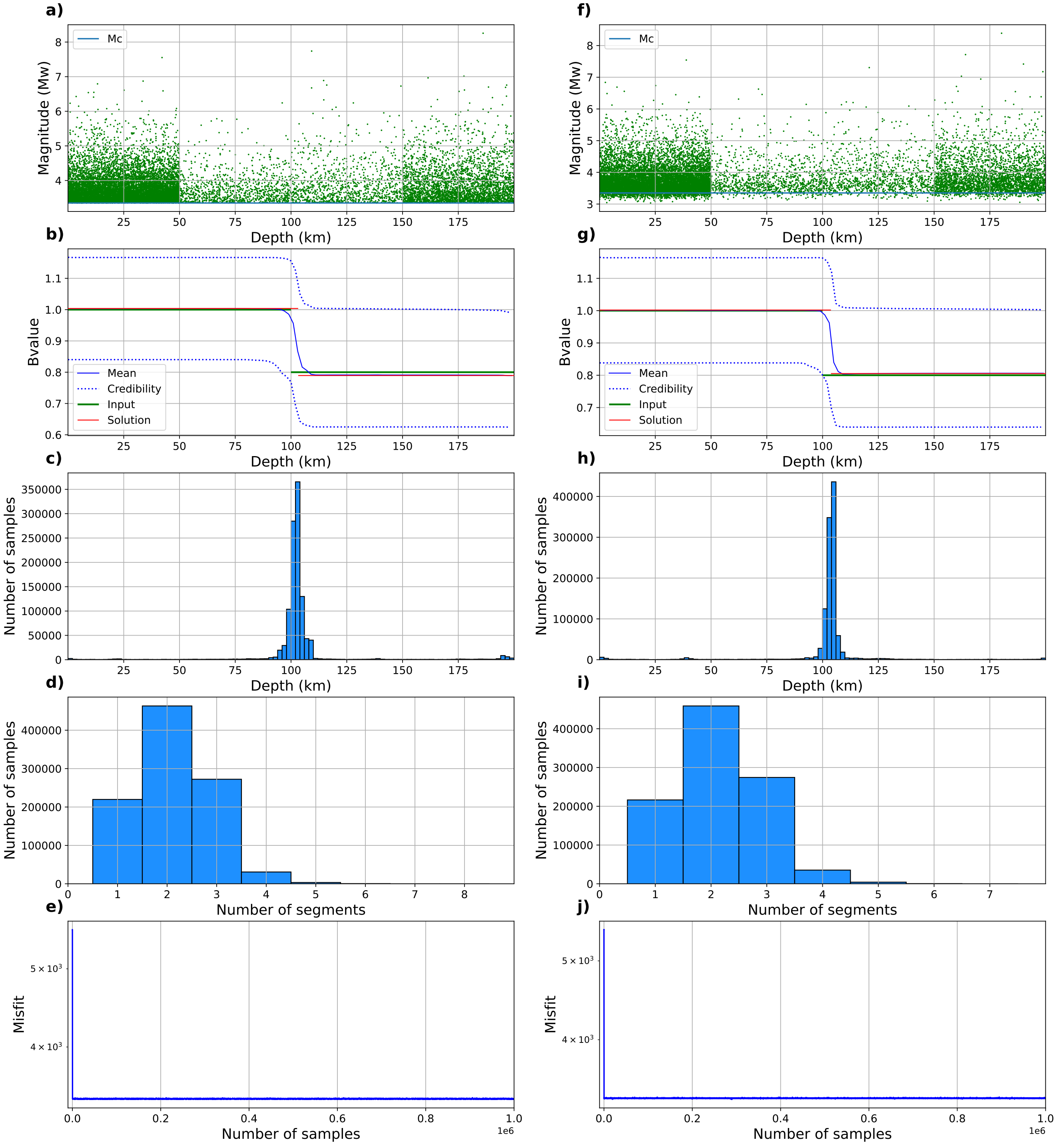

Figure S4. Summary of two synthetic tests with a scenario of four segments with a-values of 10000, 1000, 1000, and 3000 events and b-values of 1.0, 1.0, 0.8, and 0.8. The left column shows results for a noise-free catalog, and the right column noise was added to the magnitudes (standard deviation 0.1 as in the main article). a) and f) synthetic earthquake catalogs generated using two and four sets of earthquakes with different b-values. b) and g) b-value input (green), analytic solution (cyan), solutions obtained using rjMCMC: mean (blue), the best fit (red), and credibility interval (dotted blue). c) and h) Histograms that represent the amount of samples that present a change in b-value at a certain depth. d) and i) Histograms that represent the amount of samples of models with a specific number of segments. e) and j) Evolution of solution misfit.

Catalog processing

Figure S5 shows the linear regression we performed for the regional transformation of magnitudes between common events in the original microseismicity catalog and the GCMT database. We transformed all magnitudes < 6.5 from M_l to M_w. For the magnitudes > 6.5, we use the values from the GCMT database.

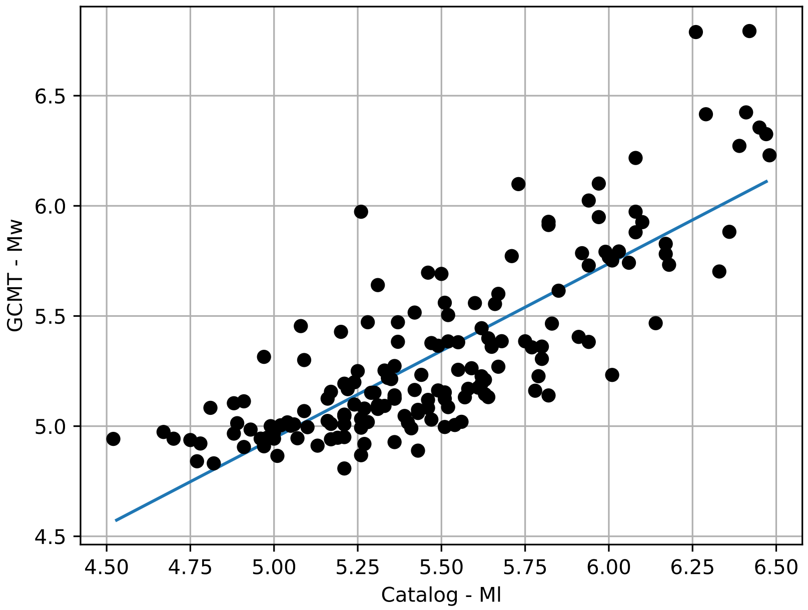

Figure S5. Linear regression of Mw vs Ml. Linear regression using the rjMCMC algorithm between the commune solutions obtained by the GCMT and our catalog.

In this work, we use the ETAS algorithm developed by Mizrahi, L. et al. (2021) to decluster the modified micro-seismicity catalog. All the codes by Mizrahi, L. et al. (2021) are open access, and are available at https://github.com/lmizrahi/etas. Inside the algorithm there is a module to compute the M_c. Before declustering we compute the completeness magnitude of the full catalog obtained the figure S4. The obtained M_c correspond to 3.35 for a b-value of 0.84.

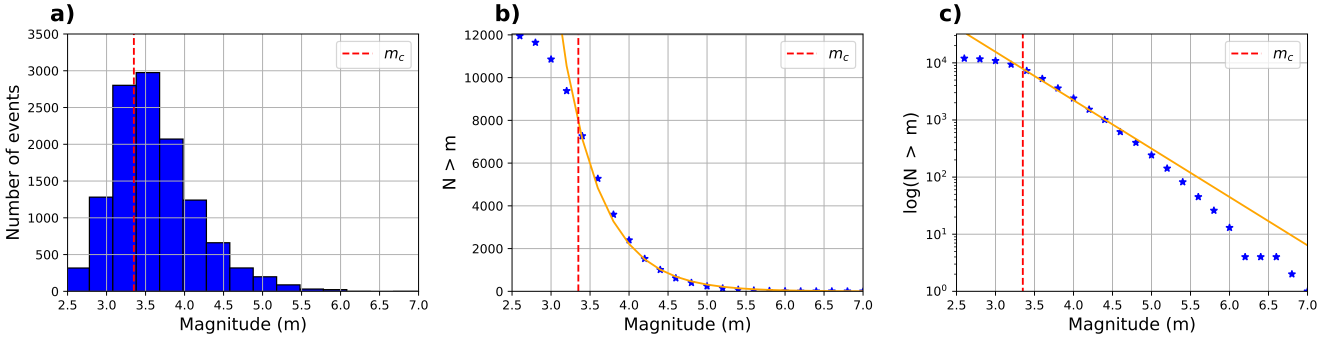

Figure S6. Solutions of completeness magnitude. Figure a) shows the frequency of the earthquakes as a function of the magnitude. b) The cumulative earthquake (blue star) and the Gutenberg-Richter equation that suit the solution (orange) and c) shows the logarithm version of b).

Declusterization procedure and sensitivity test

The declustering algorithm's output gives us the probability of each earthquake to be part of a cluster or to be an independent event. To select only independent events, we rely on the equation

$$E_{ind} = \{e_i | p_i^{ind} \leq p_{thresh})\}, \quad (1)$$

in which E_{ind} is the subgroup ($\{\}$) of independent events, formed by all i-th events e_i, given that $p_i^{ind} \leq p_{thresh})$. p_i^{ind} is the probability of each event to be an independent event and p_{thresh} is the maximum threshold such that E_{ind} contains n_{ind} events. As the declusterization problem depends on the region it is applied to, there is no sensible standard on which threshold to choose. In the present study, we choose a threshold of 0.0015 based on different trial runs and with the aim to have close to 5000 events in our final database. For further information about the algorithm, the reader is referred to the article and supplementary material of Mizrahi et al. (2021).

To test the influence of different declusterization implementations on our results, we performed the inversion of real data (results shown in Figure 4) in latitude and depth for a filtered (only events with $M > M_c$) but non-declusterized catalog as well as a filtered and over-declusterized catalog. For the latter over-declusterized catalog, we used a threshold of 0.05. The differences between the catalogs are shown in figure S6. We use all events shown in Figure S7 for the inversion with the non-declusterized catalog, and only the blue events (background seismicity) for the over-declusterized catalog.

For the non-declusterized case, figure S8 shows that the algorithm retrieves three segments along-dip, similar to the observations in the main article (Figure 4). The acceptance rate is 22%. The change points are located at 24.3 and 65.2 km with b-values of 0.88, 0.73 and 1.03 for the shallow, middle and deep segments, respectively. When over-declusterizing (Figure S9), we obtain only two segments along-dip, where the change-point is located at $\sim$60 km depth. The b-values are 0.84 for the shallower segment and 1.02 for the deeper one. The acceptance rate is 31%. This means that while the deeper changepoint appears to be robust towards the choice of events, the shallower one only appears in one case. Along strike, the solution for the non-declusterized case shows three segments, one of them very small. The acceptance rate is 23%. The change points are located at 32.02 and 31.89°S, and the b-values are 0.91, 1.21 and 0.72. Along strike, two segments can be observed, with a changepoint at 30.35°S and b-values of 0.99 and 0.64. This means that the two solutions differ from our main result, both in the number of obtained segments and the changepoint locations.

Generally, we can conclude that over- or under-declusterization has an influence on the retrieved results. Using the entire catalog can introduce artifacts e.g. from the inclusion of aftershock series with different properties than the background seismicity. Over-declusterization, in turn, leads to very low event numbers and thus very high uncertainties, which can lead to erroneous results. However, there is some consistency between the different retrieved results. They all require a changepoint in depth that is located around 60-75 km, with b-values close to 1 for the deeper segment and smaller b-values for shallower events. Likewise, the northernmost segment along-strike systematically shows the lowest b-values. As the scenarios displayed in Figures S8 and S9 can be considered extreme cases, these features should be considered highly robust.

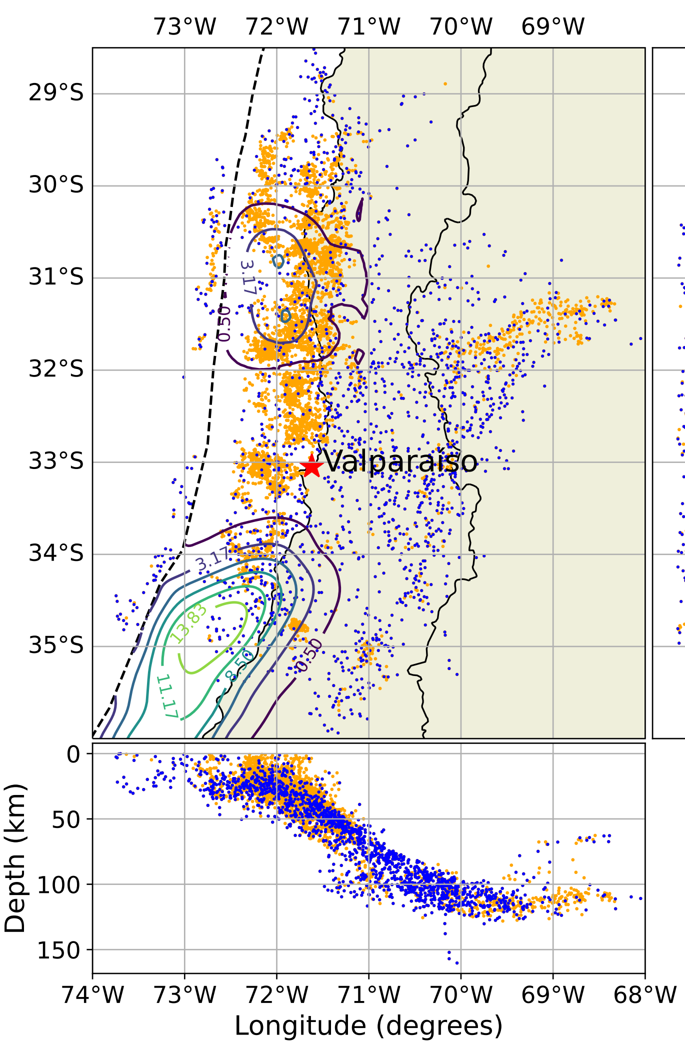

Figure S7. Spatial and temporal analysis of the seismicity. The map shows all selected events of the catalog for $M_c = 3.35$. The colors indicate background seismicity (blue) and dependent events (orange) for a choice of threshold considered as over-declusterization. The dashed line indicates the position of the trench and the solid lines show slip contours of the 2015 Illapel earthquake $M_w = 8.3$ (Tilmann et al., 2016) and the 2010 Maule earthquake $M_w = 8.8$ (Moreno et al., 2010). Red lines in the right subplot show the position in time of the 2015 Illapel earthquake and the 2017 Valparaiso earthquake $M_w = 6.9$ (Ruiz et al., 2018). This figure was created using Python 3.8.13, Matplotlib 3.5.2 and Cartopy 0.21.0

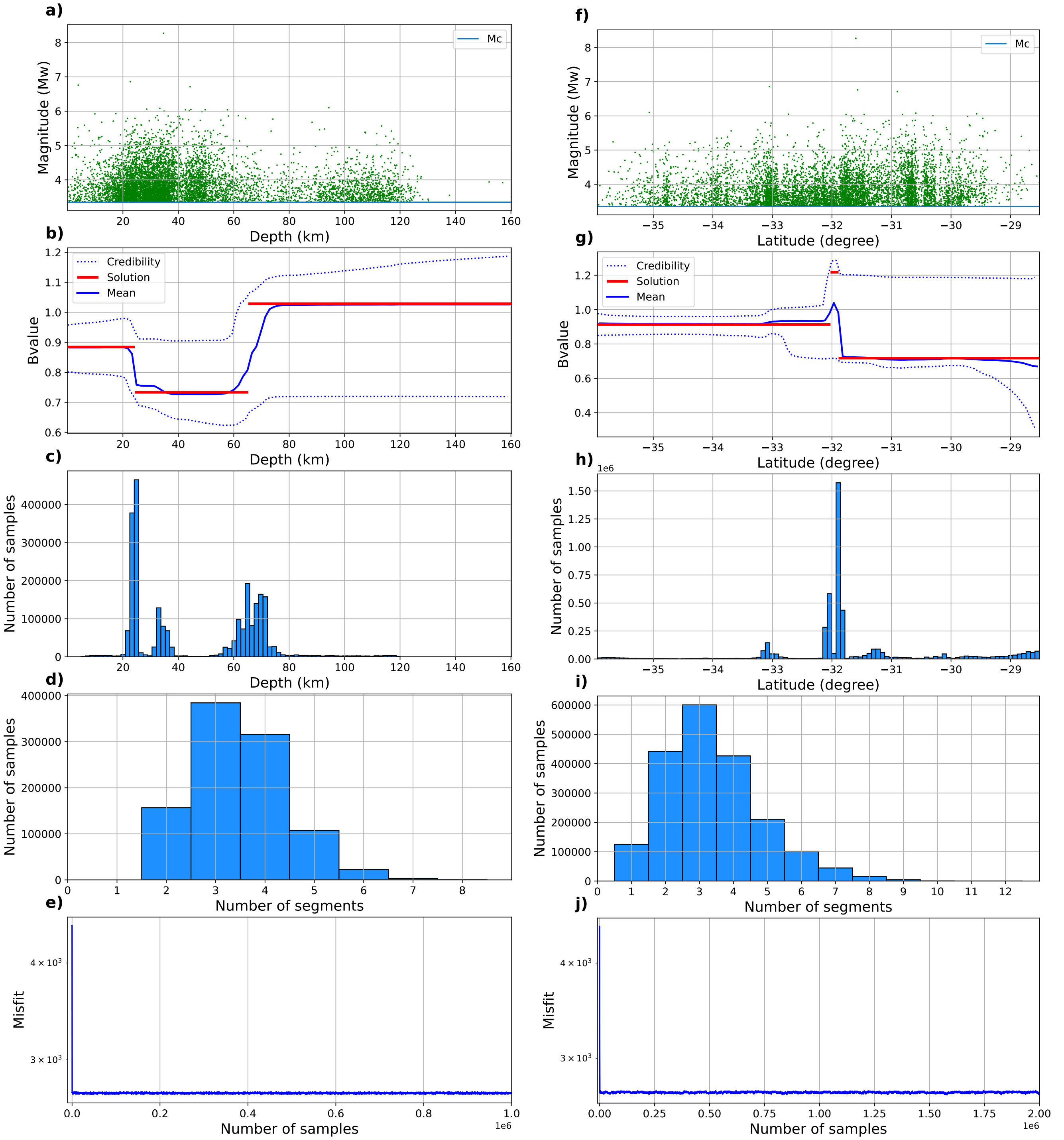

Figure S8. Inversion results for analysis of the non-declusterized catalog, with the resulting b-values as a function of depth (a-e) and latitude (f-j). a) and f) Earthquake catalog as a function of the dependent variable. b) and g) Solutions obtained using rjMCMC: mean (blue), the best fit (red), and credibility interval (dotted blue). c) and h) Histograms representing the amount of samples that show b-value changepoints at a specific depth or latitude. d) and i) Histograms representing how many samples showed each number of segments. e) and j) Evolution of solution misfit.

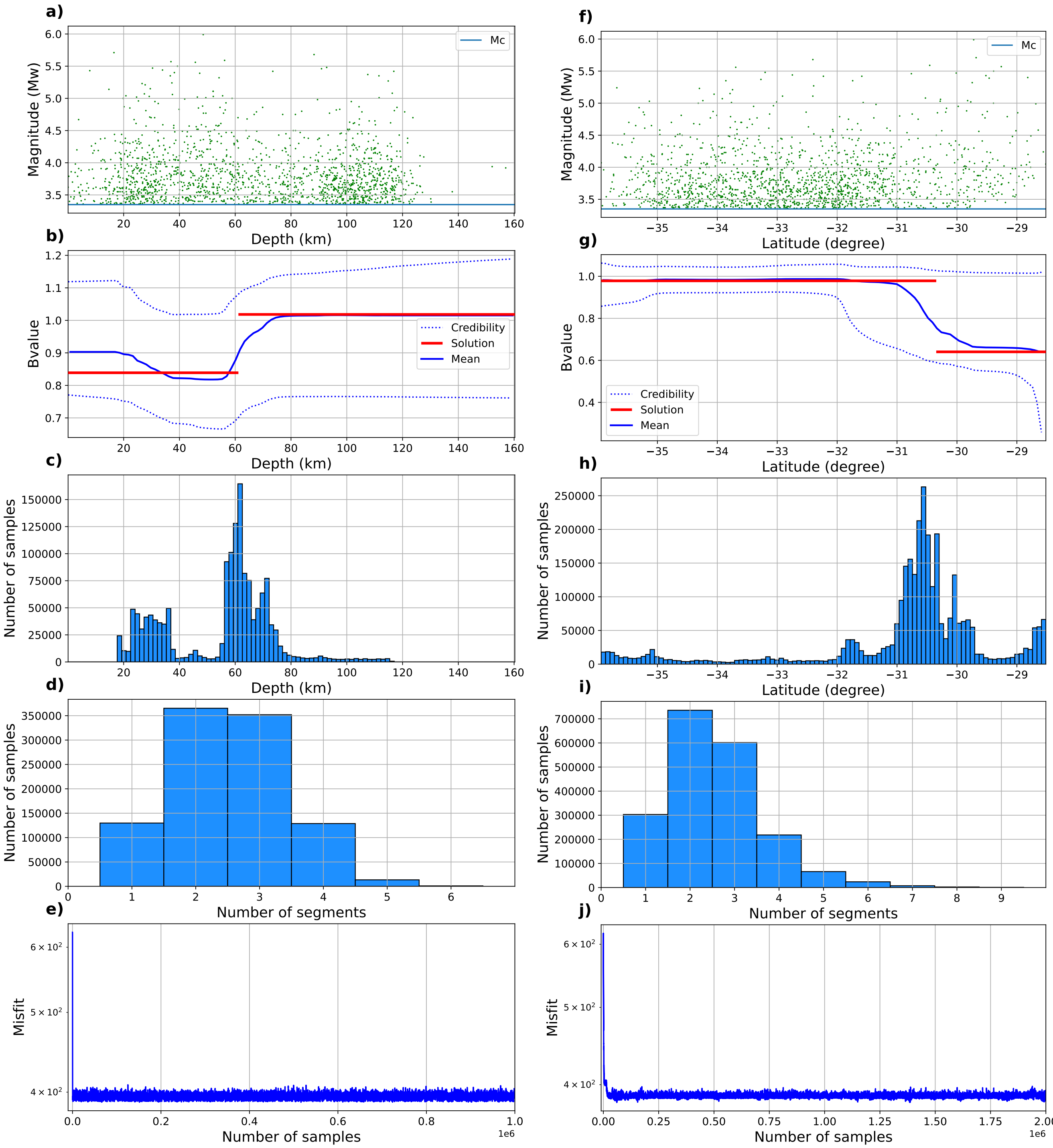

Figure S9. Inversion results for analysis of the over-declusterized catalog, with the resulting b-values as a function of depth (a-e) and latitude (f-j). a) and f) Earthquake catalog as a function of the dependent variable. b) and g) Solutions obtained using rjMCMC: mean (blue), the best fit (red), and credibility interval (dotted blue). c) and h) Histograms representing the amount of samples that show b-value changepoints at a specific depth or latitude. d) and i) Histograms representing how many samples showed each number of segments. e) and j) Evolution of solution misfit.
